# The complete mitochondrial genome of *Neomyia cornicina* (Diptera: Muscidae)

**DOI:** 10.1080/23802359.2021.1966330

**Published:** 2021-08-18

**Authors:** Zhiyun Pi, Yangshuai Jiang, Shujuan Wang, Jifeng Cai, Yadong Guo

**Affiliations:** Department of Forensic Science, School of Basic Medical Sciences, Central South University, Changsha, Hunan, China

**Keywords:** Mitochondrial genome, *Neomyia cornicina*, Muscidae, phylogenetic analysis

## Abstract

*Neomyia cornicina* (Fabricius, 1781) (Diptera: Muscidae) is considered to be an important dung-degrading species in Japan. In this study, we report the first mitochondrial genome (mitogenome) of *N. cornicina*. The complete mitogenome of *N. cornicina* was 17,254 bp in length (GenBank accession No. MW592695), containing 13 protein-coding genes (PCGs), 22 transfer RNA (tRNA) genes, 2 ribosomal RNA (rRNA) genes, and a non-coding AT-rich region. Its nucleotide composition was A (41.0%), G (8.4%), C (11.8%), and T (38.8%). Phylogenetic analysis indicated that *N. cornicina* is closely related to the species of *Eudasyphora canadiana*. This mitogenome contributes useful information for further understanding of the phylogenetic relationship and species identification within Muscidae species.

*Neomyia cornicina* (Fabricius 1781), which belongs to Muscidae, Diptera, is mainly spread in Asia and found as an important dung-degrading species in Japan (Mitsuhiro and Minori 2014). The mitogenome has been widely used for species identification of insects and estimations of evolutionary relationships in phylogenetic analyses (Shang et al. 2019). In this study, the complete mitogenome of *N. cornicina* was 17,254 bp in length, containing 13 protein-coding genes (PCGs), 22 transfer RNA (tRNA) genes, 2 ribosomal RNA (rRNA) genes, and a non-coding AT-rich region. Additionally, it displayed characteristics of A (41.0%), G (8.4%), C (11.8%), and T (38.8%).

The adult specimens of *N. cornicina* were captured in September 2020 from Qilian mountain (39°2′N, 98°5′E), Qinghai province, China. All specimens were frozen to death and then identified by forensic entomologists according to the keys of traditional morphological characteristics (Xue and Zhao 1996). These specimens were deposited in Guo’s laboratory (Department of Forensic Science, School of Basic Medical Sciences, Central South University) with a unique code separately, storing at −80 °C. The code of the specimen used in this experiment is CSU20210218. Total DNA was extracted from thoracic muscle tissues of adult specimens using the QIANamp Micro DNA Kit according to the manufacture’s instruction. The complete mitogenome was sequenced on an Illumina HiSeq 2500 Platform, and then de novo assembly was performed using MITObim 1.9.1 (https://github.com/chrishah/MITObim), and the rough boundaries of all genes were initially identified using the MITOS2 Web Server (http://mitos2.bioinf.uni-leipzig.de/index.py) (Bernt et al. 2013). The *N. cornicina* mitogenome has been submitted to GenBank with accession number MW592695.

The phylogenetic analyses of *N. cornicina* with fifteen Muscidae species were performed using the maximum likelihood (ML) method based on 13 PCGs. *Calliphora vomitoria* and *Chrysomya pinguis* (Diptera: Calliphoridae) were used as outgroups (Figure 1). ML analysis was carried out with IQ-TREE v1.6.7. The evolutionary model selected for ML analysis was GTR. The phylogenetic trees indicated that *N. cornicina* is closely related to *Eudasyphora canadiana*. This study contributes useful information for further analyzing of the phylogenetic relationships, as well as enriching the mitogenome database of Muscidae species for species identification.

**Figure 1.**
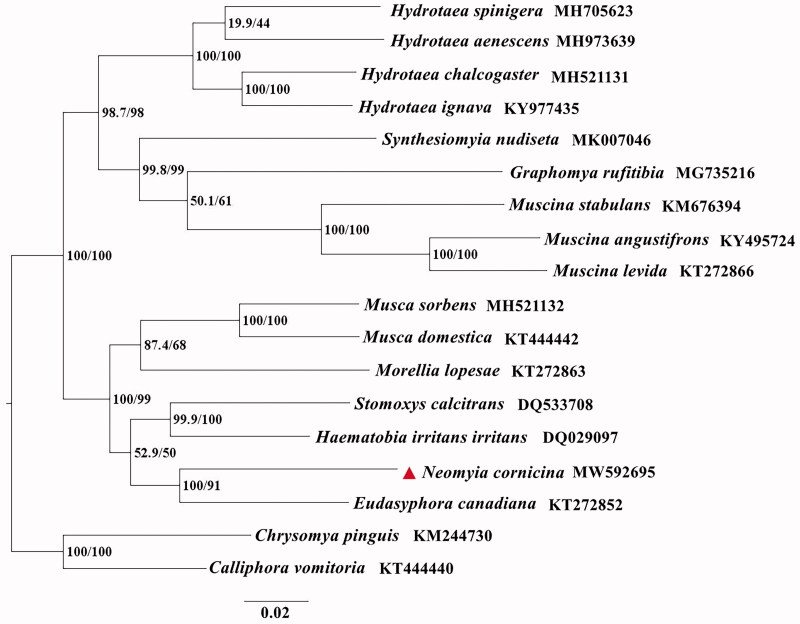
The phylogenetic trees of *N. cornicina* with fifteen Muscidae species based on 13 PCGs by using the maximum likelihood (ML) method. *Calliphora vomitoria* and *Chrysomya pinguis* (Diptera: Calliphoridae) were selected as outgroups. The indices stand for posterior probabilities/bootstrap values.

## Data Availability

The mitogenome data that support the findings of this study are openly available in GenBank of NCBI at (https://www.ncbi.nlm.nih.gov/) under the accession No. MW592695. The associated BioProject, SRA, and BioSample numbers are PRJNA703414 (https://www.ncbi.nlm.nih.gov/bioproject/PRJNA703414), SRR13757204 (https://www.ncbi.nlm.nih.gov/sra/SRR13757204), and SAMN18017668(https://www.ncbi.nlm.nih.gov/biosample/SAMN18017668), respectively. All samples were stored in Guo’s laboratory (Yadong Guo Ph.D, gdy82@126.com).
